# Targetable Pathways in the Treatment of Retroperitoneal Liposarcoma

**DOI:** 10.3390/cancers14061362

**Published:** 2022-03-08

**Authors:** Lucia Casadei, Fernanda Costas Casal de Faria, Alexandra Lopez-Aguiar, Raphael E. Pollock, Valerie Grignol

**Affiliations:** The James Comprehensive Cancer Center, Department of Surgery, Division of Surgical Oncology, The Ohio State University Wexner Medical Center, Columbus, OH 43210, USA; lucia.casadei@osumc.edu (L.C.); fernanda.costascasaldefaria@osumc.edu (F.C.C.d.F.); alexandra.lopez-aguiar@osumc.edu (A.L.-A.); raphael.pollock@osumc.edu (R.E.P.)

**Keywords:** retroperitoneal liposarcomas, MDM2, DDLPS, CDK4, miRNAs

## Abstract

**Simple Summary:**

This review discusses current and prospective treatment strategies for retroperitoneal liposarcoma, a rare type of sarcoma with a high propensity for locoregional recurrence and low survival rate. Chemo- and radiotherapy regimens, as well as molecular targets, are highlighted as important tools to better explore the mechanisms underlying this disease and to pursue new possible targetable pathways.

**Abstract:**

Liposarcoma (LPS) is the most prevalent soft tissue sarcoma histological subtype. When it occurs in the abdomen the overall survival rate is as low as 10% at 10 years and is fraught with high rates of recurrence, particularly for the more aggressive dedifferentiated subtype. Surgery remains the mainstay of treatment. Systemic therapies for the treatment of metastatic or unresectable disease have low response rates. Deep understanding of well-differentiated and de-differentiated LPS (WDLPS and DDLPS, respectively) oncologic drivers is necessary for the development of new efficacious targeted therapies for the management of this disease. This review discusses the current treatments under evaluation for retroperitoneal DDLPS and the potential targetable pathways in DDLPS.

## 1. Introduction

Soft-tissue sarcomas (STS) are a heterogenous group of tumors that represent about 1% of all adult malignancies [1]. Retroperitoneal sarcomas are a subtype of this rare disease, comprising 20% of all STS [1]. They often present late in their disease course due to non-specific symptoms such as increasing abdominal girth, abdominal pain, and change in bowel function [2,3]. Among retroperitoneal sarcomas, retroperitoneal liposarcomas (RPLPS) represent one of the most common histologic subtypes, with their own distinct biology and high risk for local versus distant recurrence [2,3]. RPLPS are morphologically classified into four subtypes: (1) well differentiated, (2) dedifferentiated, (3) myxoid, and (4) pleomorphic. Well-differentiated liposarcoma (WDLPS) and dedifferentiated liposarcoma (DDLPS) are the most common histologic subtypes. WDLPS is a typically indolent but can be locally aggressive, while DDLPS has a higher-grade histology, faster growth, and distant metastatic potential [4]. Myxoid liposarcoma is the second most common subtype, and it represents about 5% of all soft tissue sarcomas in adults [5]. Histological lesions show with low grade forms and poorly differentiated round cells. At a molecular level, translocation (12;16) (q13;p11), resulting in *FUS-DDIT3* gene fusion, has been described in the vast majority of these tumors [6]. The treatment is generally surgical excision with or without radiation therapy. In case of high-risk disease and positive surgical margins, chemotherapy is considered [7]. Pleomorphic is very rare and represents only 5% to 10% of liposarcoma [8]. However, it is considered to be of the highest malignancy grade, with high invasion, metastasis, and recurrence. Therapeutic strategies for pleiomorfic are controversial, but surgery, especially radical resection, remains the main treatment [9]. Surgical resection remains the mainstay of treatment for primary RPLPS [1], and the importance of resection with complete macroscopic clearance of tumor on recurrence-free survival (RFS) has been well established. However, the difference in outcomes between microscopically negative (R0) versus microscopically positive (R1) margins is less clear, particularly given that R1 resections are more common due to the lack of fascial planes and multiple critical structures that reside in the retroperitoneum [1,10,11,12]. As such, the typical operative approach is to resect involved organs and structures and blockages, as able, to maximize the possibility of microscopically negative margins [1]. RPLPS often require a wider margin than other retroperitoneal sarcomas histologic subtypes, such as leiomyosarcomas, since their well-differentiated component is frequently difficult to distinguish from normal retroperitoneal fat [1]. Indeed, because RPLPS have a propensity for locoregional recurrence, a thorough initial resection can affect prognosis and shape future treatment. The use of multimodality treatment, including chemotherapy and radiation to improve rates of recurrences and prognosis, has had a limited effect to date. However, histology driven treatment and clinical trials may improve on the selection of patients for these therapies. This review will be focused on the WDLPS and DDLPS (referred as RPLPS) current treatments under evaluation and the potential targetable pathways.

## 2. Current Therapies in RPLPS

### 2.1. Chemotherapy Strategies

Chemotherapy use in the adjuvant setting has not had a significant impact on recurrence and is reserved for high grade tumors with high metastatic rates. Given the hematologic mode of metastasis for RPLPS, the use of perioperative chemotherapy to theoretically target micrometastatic disease or help downsize tumors to allow a higher rate of R0 resections has been postulated [13]. However, studies have not consistently supported this theory [14,15]. Currently, anthracycline-based chemotherapy regimens, such as doxorubicin, are the first line of treatment for advanced or metastatic liposarcoma [1]. Indeed, the EORTC 62,012 trial demonstrated that liposarcomas responded better to chemotherapy than other sarcoma subtypes [16]. Second line agents such as trabectedin have also been studied, but these have been found to be primarily beneficial among the myxoid liposarcoma histologic subtype (Table 1) [17]. In 2016, the agent Eribulin was approved for use in liposarcomas/leiomyosarcomas when a phase 3 trial demonstrated a 2-month improvement in overall survival among patients treated with eribulin compared with dacarbazine [18]. The selective CDK4/CDK6 inhibitor, Palbociclib, was also shown to be associated with favorable progression-free survival in a phase 2, non-randomized trial by Dickson et al. [19]. However, the role of other anticancer agents in the treatment of RPLPS, including tyrosine kinase inhibitors or gemcitabine/docetaxel combinations, remains unclear [1].

### 2.2. Radiotherapy Strategies

The scientific literature examining the use of radiation as a treatment modality is inconsistent and largely retrospective in nature. Several studies demonstrated that higher or selective radiation dose may improve outcomes, especially for patients at high risk of local recurrence [20]. One recent phase III randomized controlled trial, the STRASS-1 trial (Surgery With or Without Radiation Therapy in Untreated Nonmetastatic Retroperitoneal Sarcoma), evaluated oncologic outcomes in patients with RPS who underwent neoadjuvant radiation followed by surgery versus surgery alone [21]. Although no statistically significant difference in RFS was seen among the two groups (3-year RFS was 60.4% in the radiation group vs. 58.7% in the surgery-only group), there was a trend suggesting that certain histologic subtypes, such as well-differentiated liposarcoma (WDLPS) and low-grade de-differentiated liposarcoma (DDLPS), may benefit from neoadjuvant radiation [1,21]. For those that advocate for its use, the benefit of administering radiation in the neoadjuvant setting is the ability to limit the exposure of abdominal viscera and vital structures to radiation toxicity due to their displacement by tumor. Currently, the Retroperitoneal Sarcoma Registry: an International Prospective Initiative (RESPAR; ClinicalTrials.gov Identifier NCT03838718) is an ongoing prospective study seeking to evaluate oncologic outcomes among patients who receive multimodality therapy, including radiation. A phase I clinical trial called Proton or Photon RT for Retroperitoneal Sarcomas (ClinicalTrials.gov Identifier NCT01659203) is also underway, and it aims to determine the highest dose of radiation therapy with protons or intensity-modulated radiation therapy that can be delivered safely in patients with RPS (Table 1).

## 3. Molecular Mechanisms as Targets for RPLPS Treatment

**Figure 1:**
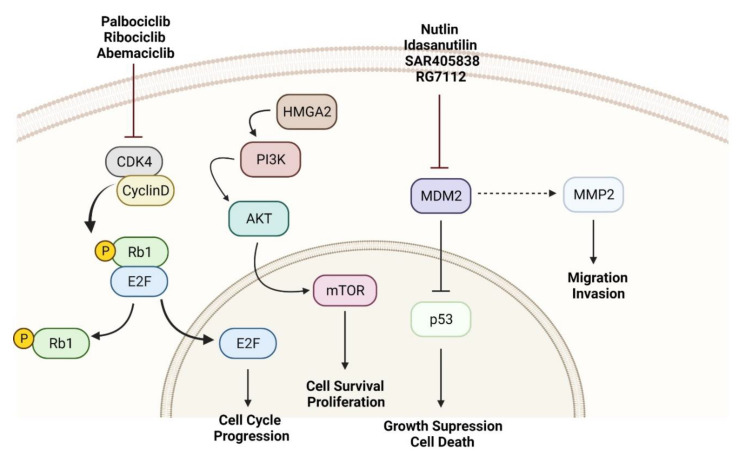
Target pathways on WD/DDLPS treatment. MDM2, CDK4, and HMGA2 amplification promote cell survival, proliferation, and metastatic phenotype in RPLPS. These pathways could be considered as targeted therapies for WD/DDLPS treatment.

RPLPS genomic profile has proved to be diverse within its subtypes. Therefore, several studies are now focused on exploring the different pathways associated with RPLPS subtypes [22]. The deeper understanding of the already known pathways, as well as the identification of novel genes and molecular mechanisms associated with RPLPS, may contribute to the development of targeted therapies (Figure 1) [15].

### 3.1. MDM2 as a Molecular Driver and Target

The main molecular characteristic of the most common RPLPS (WD-DDLPS) are genetic abnormalities (ring or giant chromosomes and double minutes) which lead to amplification of genes located on chromosome 12q13-15 such as MDM2, *CDK4*, and *HMGA2* [23,24]. Characteristic of RPLPS is also the amplification of other genes belonging to chromosome 1p32 and 6p23, such as *JUN* and *ASK1* [25]. Generally, in RPLPS, the gene tp53 is in wild-type (WT) state. Mechanistically, MDM2 protein overexpression induces inhibition of p53 and its tumor suppression function. Specifically, p53 binds MDM2 to its P2 promoter and enhances MDM2 expression. Subsequently, the high level of MDM2 protein, by binding to p53, prevents MDM2 transcription and induces proteasome-dependent degradation of p53 [26]. Therefore, the key mechanism for RPLPS growth and progression has been established to be MDM2 amplification and consequent p53 inhibition.

Since *MDM2* amplification and subsequent reduction of p53 activity has been recognized as one of the major mechanisms driving the RPLPS phenotype, targeting the MDM2-p53 axis is an attractive therapeutic strategy [27]. Several molecules have been developed to block the protein–protein interaction between p53 and MDM2. The Nutlins (Nutlin-1, -2, and -3) were the first selective and potent MDM2 inhibitors discovered, followed by RG7112, Idasanutlin, and SAR405838 [28]. These small molecules have led to the development of multiple drugs, with RG7112 serving as the first one to be clinically assessed. Unfortunately, many preclinical studies reported on-target toxicity and negative effects on lymphoid organs and the gastrointestinal tract with these drugs [29,30,31].

In addition to targeting the p53 axis, it has also been reported that MDM2 inhibitors can sensitize cells to chemotherapeutic-mediated apoptosis [32]. This supports the combination of MDM2 inhibitors with chemotherapeutic agents such as cytarabine, daunorubicin, azacytidine, decitabine, and carboplatin. There are several ongoing MDM2 inhibitors trials, however not related to WD/DDLPS. Similarly, clinical investigation of MDM2 inhibition in combination with radiation therapy is also in progress in patients with soft tissue sarcoma (NCT03217266) [33].

Another molecular mechanism through which MDM2 affects RPLPS growth is through extracellular vesicles (EVs). EVs are bilayer particles 20–100 nm in size that recently have been found to play a crucial role in the communication between the tumor cell and its microenvironment [34,35]. In RPLPS, cancer cells release EVs into the microenvironment, with *MDM2* serving as their functional cargo [36]. In particular, it has been described that *MDM2* DNA transfer from RPLPS to preadipocytes (P-a) induces activation of P-a MMP2 [36]. MMP2 is involved in one of the key initial events underlying tumor cell dissemination and recurrence: extracellular matrix degradation [37]. Indeed, MMP2 has been shown to promote cancer progression by degrading basement membrane components and collagen break down into peptides that act as chemo attractants for circulating tumor cells [38]. In the context of liposarcoma, MMP2 has been correlated with cell invasiveness, metastasis, and grade [39,40]. This new finding suggests that, although previously not considered, the RPLPS microenvironment (specifically P-a cells) may participate in RPLPS recurrence events and may be pertinent to the extremely high rate of RPLPS multifocal recurrence.

### 3.2. CDK4 Inhibitors

It is well-known that the majority of DDLPS patients will present highly amplified sequences from the 12q13-15 chromosomal region, which contains *MDM2* and *CDK4* genes [41]. In fact, WD/DD LPS are complex tumors with multiple chromosomal alterations and mutations of pivotal genes associated with oncogenesis, which is a probable explanation for the poor response to systemic chemotherapy observed in DDLPS patients [42]. Amplification and overexpression of CDK4 is generally found in WDLPS/DDLPS cells [43]. In fact, Kim and colleagues demonstrated that co-overexpression of MDM2 and CDK4 in transformed stem cells causes the blockage of adipogenic potential, leading to a high-grade sarcoma with a DDLPS-like morphology [44].

CDK4 is a cyclin-dependent kinases (CDK) that forms a complex with D-type Cyclins (CCND), thus playing an important role in cell cycle progression from G1 to S phase by allowing E2F to be released from pRb control [45]. Several CDK4 inhibitors (CDK4i) have been developed for clinical use, and their activity as single agents in the treatment of solid tumors denote CDK4 as a valid therapeutic target [46]. Palbociclib, ribociclib, and abemaciclib are some of the current CDK4i and CDK6i approved for clinical use. Their mechanism of action is based on competitive binding to and inactivation of the CDK4/CDK6 ATP pocket, with subsequent increased pRb activity [46]. As a result, CDK4 has emerged as a potential target for LPS treatment. Zhang and colleagues demonstrated that continued treatment with CDK4i as a single agent leads to decreased proliferation of DDLPS cell lines, as well as inhibited tumor growth in vivo xenograft model [47]. However, after prolonged dosing, the reestablishment of pRb phosphorylation and cell cycle progression was observed [47]. Further exploring the effects of CDK4i in LPS, Laroche-Clary and colleagues showed that the combination of palbociclib and MDM2 antagonist, RG7388, synergistically triggers proapototic and antiproliferative functions of p53 [48]. In this context, CDK4i might act as MDM2 antagonist enhancers in WDLPS/DDLPS. Clinical trials using CDK4i are currently being conducted in WDLPS/DDLPS, both as a single-agent strategy and in combination with the mTOR inhibitor, everolimus, or MDM2 antagonist, HDM201 [49,50]. As a single-agent strategy, the authors have found that abemaciclib treatment in patients with advanced progressive DDLPS results in favorable progression free survival (PFS) and objective tumor response with low toxicity [49]. Ribociclib in combination with HDM201 treatment data demonstrated a preliminary efficacy in patients with locally advanced or metastatic WDLPS/DDLPS [44].

### 3.3. Aurora Kinase Inhibitors

The Aurora kinases are a family of serine/threonine kinases that play a critical role in the G2 and M phases of the cell cycle. Aurora A kinase (AURKA) plays an important role in maintaining genomic integrity since it closely regulates centrosome assembly and proper functioning of the mitotic spindle apparatus [51]. Due to their deregulated expression in different types of tumors, these kinases have become attractive targets in cancer therapy. *AURKA* has also been found to be overexpressed in DDLPS in genomic analyses [52,53,54]. Further genomic studies have shown that *AURKA* expression is significantly higher in DDLPS compared with WDLPS and is highly correlated to metastatic recurrence [53,54]. The same studies also demonstrated a decreased number of viable cells and apoptosis induction in LPS cell lines knocked down for *AURKA* [53,54]. Taken together, these studies highlight AURKA as a potential biomarker for predicting poor prognosis and as a promising target for LPS treatment.

Since the altered expression of AURKA has been reported in liposarcomas, multiple studies have evaluated the utility of targeted Aurora kinase inhibitors [53,54,55,56]. Nair and Schwartz observed that MLN8237, a dual inhibitor of Aurora kinase A and Aurora kinase B, inhibited cellular growth in a p53 dependent manner in vitro, as well as suppressed in vivo liposarcoma tumor growth [56]. Yen and colleagues, using the same AURK inhibitor, MLN8237, demonstrated similar in vitro results. After treatment of LPS cell lines, the authors observed that MLN8237 induced G2/M arrest, exerted cytotoxic effects by causing apoptosis, and promoted synergistic outcomes when combined with chemotherapeutic agents [53]. In another study by Mattei and colleagues, the pan Aurora Kinase inhibitor, AMG 900, was evaluated as an independent drug or in combination with doxorubicin in LPS cell lines [54]. In this study, AMG 900 treatment reduced cell survival and clonogenic proliferation while simultaneously inducing apoptosis. Moreover, the combined treatment of AMG 900 with doxorubicin enhanced the effect of doxorubicin alone. By analyzing the kinome of LPS cell lines after AMG 900 treatment, the authors also found that the MAPK pathway inhibition might be linked to the effects of this pan Aurora kinase inhibitor [54]. Therefore, inhibiting Aurora kinases could be an encouraging therapy for LPS.

### 3.4. Other Kinases Associated with LPS

Receptor tyrosine kinase (RTK) genes are amplified in approximately 30% of WDLPS/DDLPS samples, and the inhibition of specific RTKs may contribute to the establishment of an effective therapeutic option for patients with LPS [57]. Indeed, a kinase profiling analysis by Kanojia et al. identified novel liposarcoma targets and probable kinase inhibitors to use as a liposarcoma treatment strategy. In this study, the screening assays revealed PTK2 and KIT as important kinases for liposarcoma cell survival and ponatinib as an effective therapeutic agent against liposarcoma cells [58]. Ponatinib is a multi-targeted tyrosine kinase inhibitor, targeting various kinases including KIT, VEGFR, PDGFR, and EGFR [59]. In vitro analysis has shown reduced clonogenic proliferation, apoptosis induction, and cell cycle arrest at the G0/G1 phase in LPS cell lines after ponatinib treatment. These effects were mediated by a decrease in KIT phosphorylation and its downstream signaling pathway. In addition, liposarcoma tumor growth in a xenograft model has also been shown to be inhibited by ponatinib [58].

Pazopanib, another multi-targeted tyrosine kinase inhibitor against VEGFR and KIT, significantly decreases tumor growth and inhibits angiogenesis in liposarcoma xenografts models [60]. Despite some clinical trials demonstrating insufficient tumor response, the use of pazopanib in treating intermediate and high-grade liposarcoma shows mostly promising results [61,62]. One postulated theory for the discrepancies observed may be related to intratumoral heterogeneity, thus leading to an underestimation of individual genomic alterations [57,62].

### 3.5. Immune Checkpoint Inhibitors

The programmed cell death-1 (PD-1) checkpoint pathway is an important target for immunotherapy. Immune cells such as activated T cells, monocytes, B cells, natural killer, and dendritic cells express PD-1. Since tumor cells can express the ligand PD-L1, they can modulate immune cell activity by interacting with PD-1, thus leading to an effective immune-evasion strategy [63]. Yan and colleagues provided a profile of immune characteristics of RPLPS. The authors demonstrated that T cells are more prevalent than B cells, and PD-L1 expression increases as the tumor progresses. In general, patients with higher PD1 or PD-L1 expression have a worse prognosis, and RPLPS tumors exhibit immune heterogeneity [64]. In another study, key immune-related prognostic variables and their correlation with anti-PD1 therapy were identified using a xenografted model with dedifferentiated liposarcoma. DDLPS tumors presented a significant increase in CD8+ T cell abundance, followed by a near-significant increase in activated NK cell abundance [65]. Moreover, Choi et al. demonstrated an anti-tumor effect of pembrolizumab in humanized DDLPS xenograft mice, and that effect was associated with the abundance of hCD8+ T and hNK subsets [65].

Ultimately, the clinical activity of nivolumab or pembrolizumab is dependent on the histological subtype of sarcoma, disease setting, and the combined treatment strategy. The combination of immune checkpoint inhibitors with chemotherapy seems a promising strategy for liposarcoma [66].

Immune checkpoint inhibitors have also been studied in the setting of advanced liposarcoma. However, only pembrolizumab treatment has been shown to have promise for DDLPS patients to date. In a phase II trial with pembrolizumab (SARC028), 20% of patients with DDLPS had an objective response to immunotherapy. Based on these reports, DDLPS patients may benefit from immune checkpoint inhibitor therapy, though further study is certainly needed [67,68,69].

### 3.6. miRNAs and RPLPS Microenvironment

MiRNAs are 19–24 nucleotide-long, single-stranded RNAs regulating transcription and translation of protein-coding genes [70]. MiRNA expression profiling was shown to be associated with tumor classification and stages with high sensitivity compared with conventional methodologies [71]. miRNAs can be retrieved in the bloodstream incorporated in extracellular vesicles or cell-free, and they can also be retrieved in other biological fluids such as saliva and urine [72]. The study of miRNAs in miRNAs, the most common RPLPS subtype (WD-DDLPS), has recently gained more attention. When comparing miRNAs in formalin-fixed paraffin-embedded LPS and adipose tissue samples, miR-155 and −21 were found to be upregulated in LPS samples [73,74]. Specifically, in RPLPS, high expression of miR-155 and −26a-2 has been associated with a poor prognosis [74,75]. miR-1246, −4532, −4454, −619-5p, and −6126 have been recently identified as potential serum biomarkers for RPLPS [76]. miR-25-3p and −92a-3p are highly expressed in peripheral blood plasma vesicles derived from human RPLPS patient samples. These two miRNAs were also found to increase IL-6 secretion through ligation of TLR7/8, and consequent activation of NF-κB, in macrophages [77]. This was established by dosage of IL-6 in peritoneal macrophages treated with RPLPS derived EVs or synthetic vesicles containing miR-25–3p and miR-92a-3p. Furthermore, genetically modified HEK-293 cells overexpressing human TLR8 receptor have been used to determine the participation of NF-κB to the induction of IL-6 secretion. It was shown that in macrophages, the NF-κB pathway was activated consequently to the treatment of HEK-293 overexpressing TLR8 with RPLS-derived EVs or synthetic vesicles containing miR-25-3p and miR-92a-3p [77]. This same study also showed that the increase of IL-6 was able to induce RPLS growth showing increase proliferation, migration, and invasion in consequence to RPLS cells treatment with macrophage-conditioned medium recovered after incubation with RPLS-EVs or synthetic EVs containing miR-25–3p and miR-92a-3p [77]. These data brought a new understanding of RPLS, proposing that EVs could be substantial in RPLS progression and in the communication between RPLS and its microenvironment.

## 4. Conclusions and Future Directions

Since RPLPS therapies based on the most common dysregulated molecular pathways have not demonstrated adequate treatment response, new exploration of the molecular mechanisms underlying this disease have been pursued. While the difference in clinical manifestation of the two main sarcoma histologic subtypes, WDLPS and DDLPS, is well-known, the molecular explanation for these differences remains undetermined. Much remains to be learned about the molecular implications of RPLPS genetic amplifications. Eventually, a full characterization of this rare disease at the genomic and proteomic level is anticipated. In the interim, basic pathobiological studies are necessary if new possible targetable pathways for treatment are to be discovered.

## Figures and Tables

**Table 1:** Recent and ongoing trials in retroperitoneal liposarcoma.

Trial Name	Status	Primary Aims/Findings
Retroperitoneal Sarcoma Registry: an International Prospective Initiative—NCT03838718	Recruiting	- To prospectively collect standardized clinical/radiologic/pathologic data from primary RPS treated with surgery and to evaluate patient outcomes - Sarcoma subtype: Any RPS
Proton or Photon RT for Retroperitoneal Sarcomas—NCT01659203	Recruiting	- To determine the maximum tolerated dose of preoperative IG-IMPT or IG-IMRT with boost to the high-risk margin of RPS and to determine local control rate after protocol treatment followed by surgery - Sarcoma subtype: Any RPS
Navtemadlin and Radiation Therapy in Treating Patients with Soft Tissue Sarcoma—NCT03217266	Recruiting	- Maximum tolerated dose/recommended phase 2 dosage - Sarcoma subtype: Grades 2 or 2 STS
Neoadjuvant Chemotherapy and Retifanlimab in Patients with Selected Retroperitoneal Sarcomas (TORNADO)—NCT04968106	Not yet recruiting	- Assessment of the antitumor activity of retifanlimab in association with neoadjuvant doxorubicin + ifosfamide - Sarcoma subtype: Any RPS
Surgery With or Without Neoadjuvant Chemotherapy in High-Risk Retroperitoneal Sarcoma (STRASS2)—NCT04031677	Recruiting	- Assess DFS among the preoperative chemotherapy and surgery arm versus the surgery alone arm - Sarcoma subtype: High-risk LMS or DDLPS
Preoperative Ultra-hypofractionated Radiotherapy Followed by Surgery for Retroperitoneal Sarcoma—NCT05224934	Recruiting	- Evaluate peri-operative complications - Sarcoma subtype: Any RPS
Nivolumab and BO-112 Before Surgery for the Treatment of Resectable Soft Tissue Sarcoma—NCT04420975	Recruiting	- To explore the safety of BO-112 in combination with nivolumab in STS patients undergoing preoperative radiotherapy - Sarcoma subtype: Any STS of extremity, trunk, or RP
Treatment of Milademetan Versus Trabectedin in Patient With Dedifferentiated Liposarcoma (MANTRA)—NCT04979442	Recruiting	- Compare PFS between the milademetan treatment arm and trabectedin control arm - Sarcoma subtype: DDLPS
SARC041: Study of Abemaciclib Versus Placebo in Patients With Advanced Dedifferentiated Liposarcoma—NCT04967521	Recruiting	- To determine PFS among patients treated with abemaciclib versus placebo - Sarcoma subtype: DDLPS
Palbociclib and INCMGA00012 in People With Advanced Liposarcoma—NCT04438824	Recruiting	- To confirm the recommended phase 2 dose and best overall response rate - Sarcoma subtype: WD/DDLPS
Phase II Trial of Ribociclib and Everolimus in Advanced Dedifferentiated Liposarcoma (DDL) and Leiomyosarcoma (LMS)—NCT03114527	Recruiting	- To evaluate PFS among patients treated with ribociclib in combination with everolimus - Sarcoma subtype: LMS or DDLPS
Retroperitoneal Soft-Tissue Sarcomas—NCT05044624	Completed (6/2021)	- To evaluate the effect of clean surgical margins on recurrence - Results: Pending - Sarcoma subtype: Any RPS
Surgery With or Without Radiation Therapy in Untreated Nonmetastatic Retroperitoneal Sarcoma (STRASS)—NCT01344018	Completed (1/2018)	- To evaluate the impact of preoperative radiotherapy plus surgery versus surgery alone on abdominal RFS - Results: RT should not yet be considered standard of care in treatment of RPS - Sarcoma subtype: Any RPS

RPS: Retroperitoneal sarcoma; RT: Radiotherapy; IG-IMPT: Image Guided Intensiy Modulated Proton Radiation Therapy; IG-IMRT: Image Guided Intensity Modulated Photon Radiation Therapy; DFS: Disease-free survival; PFS: Progression-free survival; RFS: Recurrence-free survival; STS: Soft Tissue Sarcoma; LMS: Leiomyosarcoma.
